# Feasibility of Smartphone‐Derived Short‐Interval Walking Tests for Monitoring Functional Recovery After Spine Surgery

**DOI:** 10.1002/jsp2.70173

**Published:** 2026-03-24

**Authors:** J. Puhakka, M. Jung, T. Fekete, M. Loibl, F. Kleinstück, F. Porchet, M. Ropelato, M. Gocevic, F. Galbusera, A. Cina, D. O'Riordan, J. Kosola, D. Haschtmann

**Affiliations:** ^1^ Department of Spine Surgery Schulthess Klinik Zürich Switzerland; ^2^ Department of Orthopaedic Surgery and Traumatology, Inselspital, Bern University Hospital University of Bern Bern Switzerland; ^3^ Department of Teaching Research and Development, Schulthess Klinik Zürich Switzerland; ^4^ Department of Orthopaedics and Traumatology University of Helsinki Helsinki Finland

## Abstract

**Background:**

Traditional assessments of functional recovery after spine surgery rely on patient‐reported outcomes, which are prone to bias. Wearables and smartphone activity tracking offer objective monitoring but may be unreliable if devices are not carried continuously. Capacity‐oriented measures, such as the 1‐min walk test (1MWT) and 6‐min walk test (6MWT), may be more reliable. This study evaluated smartphone‐derived interval metrics after lumbar spine surgery retrospectively.

**Methods:**

iPhone Health exports from 41 patients were analyzed. A sliding‐window algorithm parsed daily distances to simulate 1MWT and 6MWT. Step counts and active time were extracted. Activity was compared across four intervals: 6‐month baseline, final 2 weeks preoperatively, early postoperative (0–2 weeks), and late postoperative (2–6 weeks). Paired *t*‐tests or Wilcoxon signed‐rank tests were used, with Simes–Hochberg adjustment for multiple comparisons. Day‐to‐day stability was summarized by the coefficient of variation (CV). Pearson correlations were calculated.

**Results:**

Median 1MWT fell from 98 m at baseline to 82 m in the final two preoperative weeks (*p* < 0.05) and increased to 105 m by late recovery (*p* < 0.05 vs. preoperative). Median 6MWT declined from 403 to 345 m preoperatively, with this decline not reaching significance (*p* = 0.07), and increased to 407 m by late recovery (*p* < 0.05 vs. preoperative). Steps declined from 5030 to 3825 preoperatively (*p* < 0.05) and rose to 5538 at 2–6 weeks (*p* < 0.05 vs. preoperative). The 1MWT and 6MWT were strongly correlated. CV was lower for 1MWT and 6MWT than for steps.

**Conclusions:**

Smartphone‐derived 1MWT and 6MWT improved significantly from the immediate preoperative period to late postoperative recovery, showed lower day‐to‐day variability than longitudinal activity metrics, and were strongly correlated with each other. These findings support smartphone‐derived interval metrics as a feasible method to monitor recovery following lumbar spine surgery.

## Graphical abstract

In a retrospective analysis of iPhone Health exports from 41 patients undergoing lumbar decompression or discectomy, smartphone‐derived 1‐min walk tests (1MWT), 6‐min walk tests (6MWT), daily steps, and active time were analyzed from 6 months before surgery to 6 weeks after surgery. The 1MWT and 6MWT showed lower day‐to‐day variability than daily steps or active time, were strongly correlated with each other, declined before surgery, remained low during the first two postoperative weeks, and improved during postoperative weeks 2–6, supporting their use for monitoring functional recovery after lumbar spine surgery.

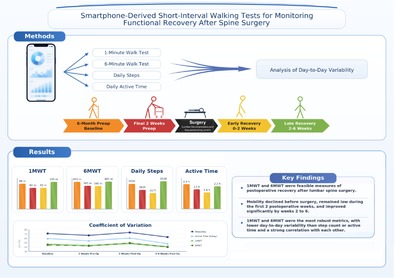

## 1 Introduction

The assessment of functional recovery in spine surgery patients has historically relied on subjective measures typically recorded as scores in so‐called patient‐reported outcome measures (PROMs), such as the Oswestry Disability Index (ODI) or the Core Outcome Measures Index (COMI) [1, 2, 3]. These measures capture and quantify the patient perspective on functional outcome domains, reflecting what a patient believes they can do. However, PROMs extend beyond the assessment of functionality alone; they also evaluate disability in a broader sense and quality of life, including the patient's perception of their condition and limitations. As highlighted by Haddas et al. and supported by previous findings [4, 5, 6], PROMs offer limited insights into objective physical function because they do not directly quantify functional capacity under standardized conditions or actual performance in daily life. PROMs are further limited by various biases [7], such as recall bias and participation‐related influences, including the Hawthorne effect, where patients modify their behavior when they are aware of being observed, as described by Demetriou et al. [8]. While motion‐based measurements do not capture these broader dimensions, they provide a more objective and precise assessment of physical function and can be seen as a valuable complement, as demonstrated by Basil et al. [9].

Unlike PROMs, objective functional assessments like the 6‐min walk test (6MWT), the Self‐Paced Walk Test (SPWT), and the Timed Up and Go (TUG) test evaluate the capacity domain, representing what a patient is capable of achieving under controlled, standardized conditions [10, 11, 12]. Furthermore, real‐world performance data recorded with smartphones or wearables capture daily activity measures such as step counts, heart rate variability, and activity duration, reflecting what a patient genuinely performs in their everyday environment [4, 13]. These three domains of physical function—patient‐reported, capacity, and performance—are distinct yet complementary in their ability to assess recovery comprehensively [4].

Advancements in wearable and mobile technologies have facilitated studies that objectively track activity in real‐world settings. Stienen et al. [14] validated the use of consumer‐grade accelerometers for activity tracking in spine surgery patients, demonstrating their feasibility and reliability.

Analysis of activity data from spine surgery patients has revealed consistent recovery patterns, including decreased activity as disease burden intensifies, low activity metrics immediately post‐surgery, and gradual improvement over the recovery period [6, 14, 15, 16, 17, 18, 19]. While daily step counts remain a popular metric, they are influenced by external factors such as environmental conditions and lifestyle, potentially obscuring subtle changes in functional capacity. Therefore, shorter interval‐based measures, such as simulated 1‐min walk tests (1MWT) and 6MWT derived from smartphone data, can be interpreted as an approximation of a patient's actual movement capacity and as a more sensitive indicator of recovery dynamics. Previous studies established the reliability of the 6MWT using smartphone applications and provided normative data for healthy [20, 21, 22] and operated patient populations, reinforcing the utility of interval‐based measures [14].

In this retrospective study, activity data exported from patients' iPhones (Apple Inc., Cupertino, CA, USA) were analyzed following lumbar decompression or discectomy. Patients undergoing these procedures were chosen because they are common treatments for degenerative lumbar pathologies in which walking impairment is a key complaint and clinically relevant improvement in mobility is typically expected within the first postoperative weeks [14, 15, 18].

By focusing on shorter activity intervals alongside daily step counts captured in a real‐world setting, we aimed to better understand how these metrics reflect recovery dynamics.

## 2 Methods

This study was conducted in accordance with ethical principles outlined by the Declaration of Helsinki and approved by the Kantonale Ethikkommission Zürich. The inclusion period spanned December 2023 to December 2024. A cohort of 41 consecutive patients who had undergone lumbar decompression surgery or discectomy/sequestrectomy was identified and recruited postoperatively at their 6‐week follow‐up. This allowed the elimination of the Hawthorne effect by utilizing previously collected smartphone data in patients' natural environments, reducing observer and performance biases. Inclusion criteria mandated the use of an iPhone both pre‐ and postoperatively to ensure continuous activity monitoring. Written informed consent was obtained from all participants prior to data transfer. Phone‐carrying habits were not assessed by questionnaire or interview.

Postoperative care followed the institutional standardized pathway. All patients received postoperative education on activity precautions for the first six postoperative weeks. Outpatient physiotherapy was part of standard care and was typically initiated around postoperative week 4.

Data were exported directly from each patient's iPhone Health application (app) using the built‐in export function. The exported files were transferred securely to the research team using AirDrop technology. This process ensured the integrity and confidentiality of patient data throughout the export and transfer process. Subsequently, the datasets were anonymized and assigned a unique personal identification number. The corresponding key linking these identification numbers to patient identities is securely stored within the hospital's internal information system.

Data analysis was blinded and conducted using a custom‐designed Python script developed for this study. The script was specifically engineered to extract, format, and analyze physical activity data from the iPhone Health app exports, focusing on walking distances, step counts, and daily active time.

### 2.1 6‐Minute and 1‐Minute Walk Test Simulation

For each day, the script simulated a 6MWT and a 1MWT by analyzing walking/running distance records from the Health app. The simulation was based on:

1. *Data parsing*: Extracting time‐stamped distance data from the XML export file.
2. *Normalization*: Distributing walking distances to a continuous timeline to create a consistent dataset where each minute in day has a value of the distance covered.
3. *Sliding window analysis*: Identifying the maximum distance covered over any 6‐min and 1‐min periods during the day using a sliding window approach from the normalized dataset.

The maximum daily distances for the 6MWT and 1MWT were recorded for subsequent analysis.

### 2.2 Daily Step Count and Active Time Analysis

The script analyzed total daily step counts by extracting and aggregating step count data by date. Additionally, the script calculated daily walking or running active time by summing the duration of all these activity periods recorded in the iPhone Health app. Active time was expressed in hours per day.

### 2.3 Statistical Analysis

To assess differences in activity, we conducted pairwise comparisons across four prespecified intervals: preoperative baseline (6 months preceding surgery), preoperative last 2 weeks, early postoperative (0–2 weeks after surgery), and late postoperative (2–6 weeks after surgery).

For each comparison, normality of the paired differences was evaluated using the Shapiro–Wilk test (*α* = 0.05). If the distribution of differences did not deviate significantly from normality (*p* ≥ 0.05), a paired Student's *t*‐test was applied. If normality was violated (*p* < 0.05), the Wilcoxon signed‐rank test was used.

To quantify the day‐to‐day stability of each metric within each interval, we calculated the coefficient of variation (CV) per patient and period as CV (%) = 100 × SD_daily/mean daily. Daily values comprised the 1MWT and 6MWT, the total daily step count, and the daily total of active time.

To control the family‐wise error rate, adjusted *p*‐values were computed using the Simes–Hochberg step‐up procedure. Statistical significance was defined as adjusted *p* < 0.05. Descriptive summaries are reported as median with interquartile range (IQR) for consistency across outcomes.

All analyses were performed using a Python script incorporating the libraries pandas, matplotlib, seaborn, scipy, and numpy for data analysis and visualization.

Artificial intelligence‐based tools were used to improve language and clarity during manuscript preparation and to assist with drafting portions of the Python code used for data processing and statistical analyses. All study design, data handling, statistical analyses, interpretation, and conclusions were performed and verified by the authors, who reviewed and approved the final manuscript.

## 3 Results

### 3.1 Demographics

A total of 41 patients (*n* = 41) were included in this study, with a mean age of 60.0 ± 16.9 years. Twnty‐five patients underwent sequestrectomy, while 16 underwent decompression procedures (Table 1).

**TABLE 1:** Demographic information.

Characteristic	Result
Age and BMI	
Age in years	60 (SD = 16.9)
BMI in kg/m^2^	26 (SD = 3.6)
Sex	
Female	18 (43.9%)
Male	23 (56.1%)
Smoking status	
Active smoker	9 (22%)
Not active smoker	32 (78%)
Diagnosis	
LDH	23 (56.1%)
LSS	4 (9.8%)
LSL + LSS	9 (22%)
LSL + LSS + LDH	3 (7.3%)
FJC	2 (4.9%)
Surgical procedure	
Lumbar discectomy	25 (61%)
Lumbar decompr.	16 (39%)
ASA risk scale	
1	9 (22%)
2	27 (65.9%)
3	5 (12.2%)
Levels treated	
1	32 (78%)
2	8 (19.5%)
3	1 (2.4%)

Abbreviations: ASA, American Society of Anesthesiologists; BMI, body mass index; FJC, facet joint cyst; LDH, lumbar disc herniation; LSL, lumbar spondylolisthesis; LSS, lumbar spinal stenosis.

### 3.2 Postoperative Care and Perioperative Events

Outpatient physiotherapy was initiated around postoperative week 4 in 36 patients. Two patients did not receive outpatient physiotherapy, and three patients started physiotherapy after postoperative week 6. Two patients attended inpatient rehabilitation after discharge, and one patient completed a convalescence stay. Two patients had an intraoperative dural tear that was repaired and managed with 1 day of postoperative bed rest without subsequent symptomatic complications. One patient had a prolonged hospital stay due to delayed wound healing. No infections occurred, and no revision surgery was required within the observation period (Table 2).

**TABLE 2:** Postoperative care and perioperative events.

Postoperative care and events	Value
Length of stay	3 (IQR 3–4), range 2–6 days
Outpatient physiotherapy initiated ~week 4	36
Inpatient rehabilitation/convalescence stay	3
Intraoperative dural tear	2
Delayed wound healing	1
Infection	0
Revision surgery (within observation)	0

### 3.3 Daily Active Time

Daily active time, defined as the duration of motion while carrying a smartphone, averaged a median of 2.4 h (interquartile range [IQR], 1.5–3.1) during the preoperative baseline period. This decreased to 1.9 h (IQR, 1.1–2.6) in the final 2 weeks before surgery and further to 1.6 h (IQR, 0.8–2.2) in the early postoperative period. By the late postoperative period, daily active time had returned to 2.2 h (IQR, 1.3–3.4). Differences across the four intervals were not statistically significant.

### 3.4 1‐Minute Walk Test

**FIGURE 1:**
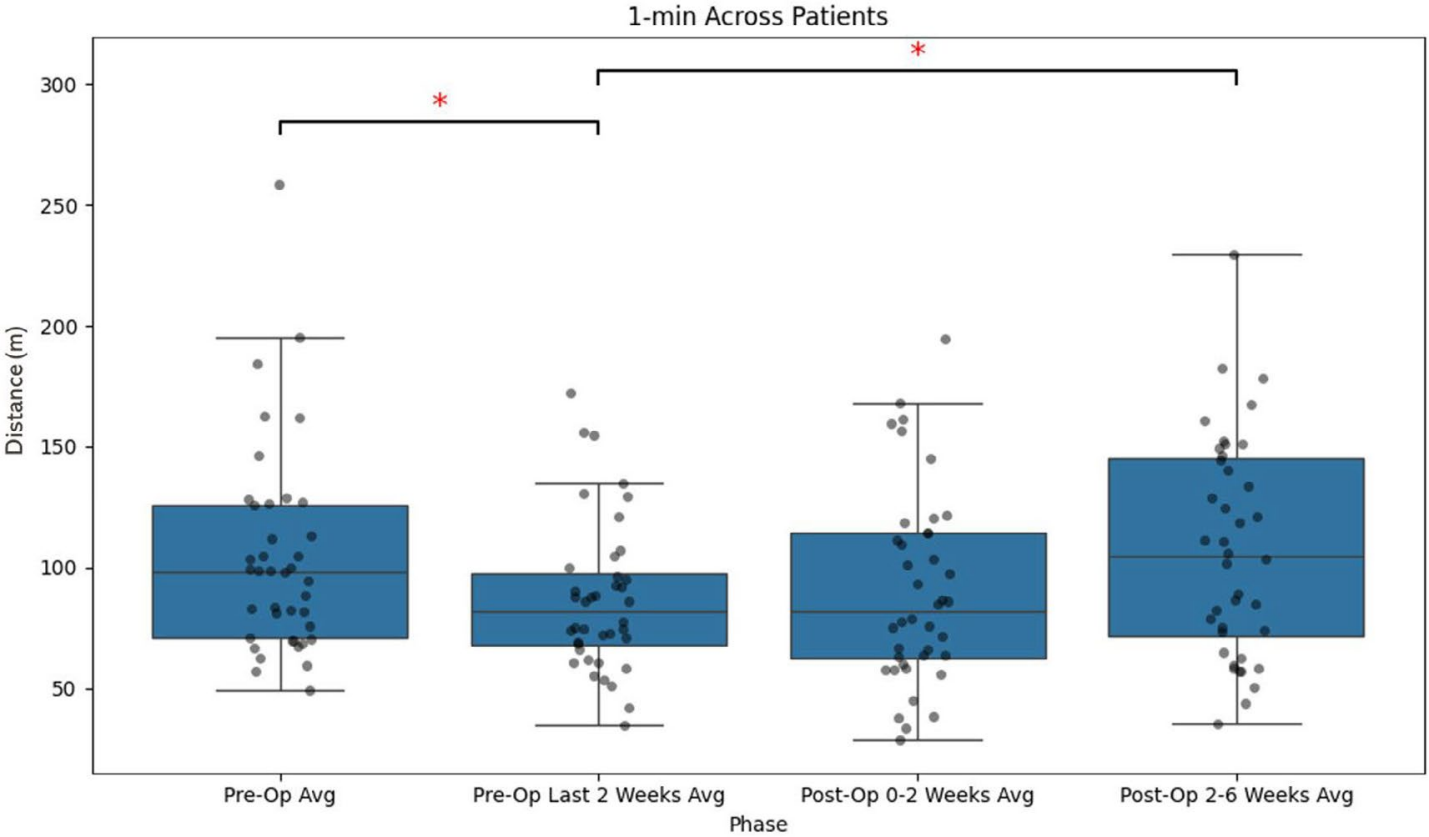
Boxplot showing the distribution of maximum daily distances in the 1MWT across different time periods. The box represents the interquartile range (IQR), with the lower and upper edges corresponding to the 25th and 75th percentiles, respectively. The horizontal line within the box indicates the median. Whiskers extend to the most extreme data points within 1.5 times the IQR from Q1 and Q3, while individual dots represent outliers beyond this range. * indicates statistical significance (*p* < 0.05). Distance (*m*) represents iPhone‐estimated walking/running distance in meters.

The 1MWT demonstrated a preoperative median of 98 m (IQR, 71–126), which declined significantly to 82 m (IQR, 68–97) in the final 2 weeks before surgery. In the early postoperative period (0–2 weeks), performance showed partial recovery (82 m; IQR, 62–114), with no significant difference from the preoperative last 2 weeks. By the late postoperative period (2–6 weeks), full recovery was observed (105 m; IQR, 71–145; *p* < 0.05; Figure 1).

### 3.5 6‐Minute Walk Test

**FIGURE 2:**
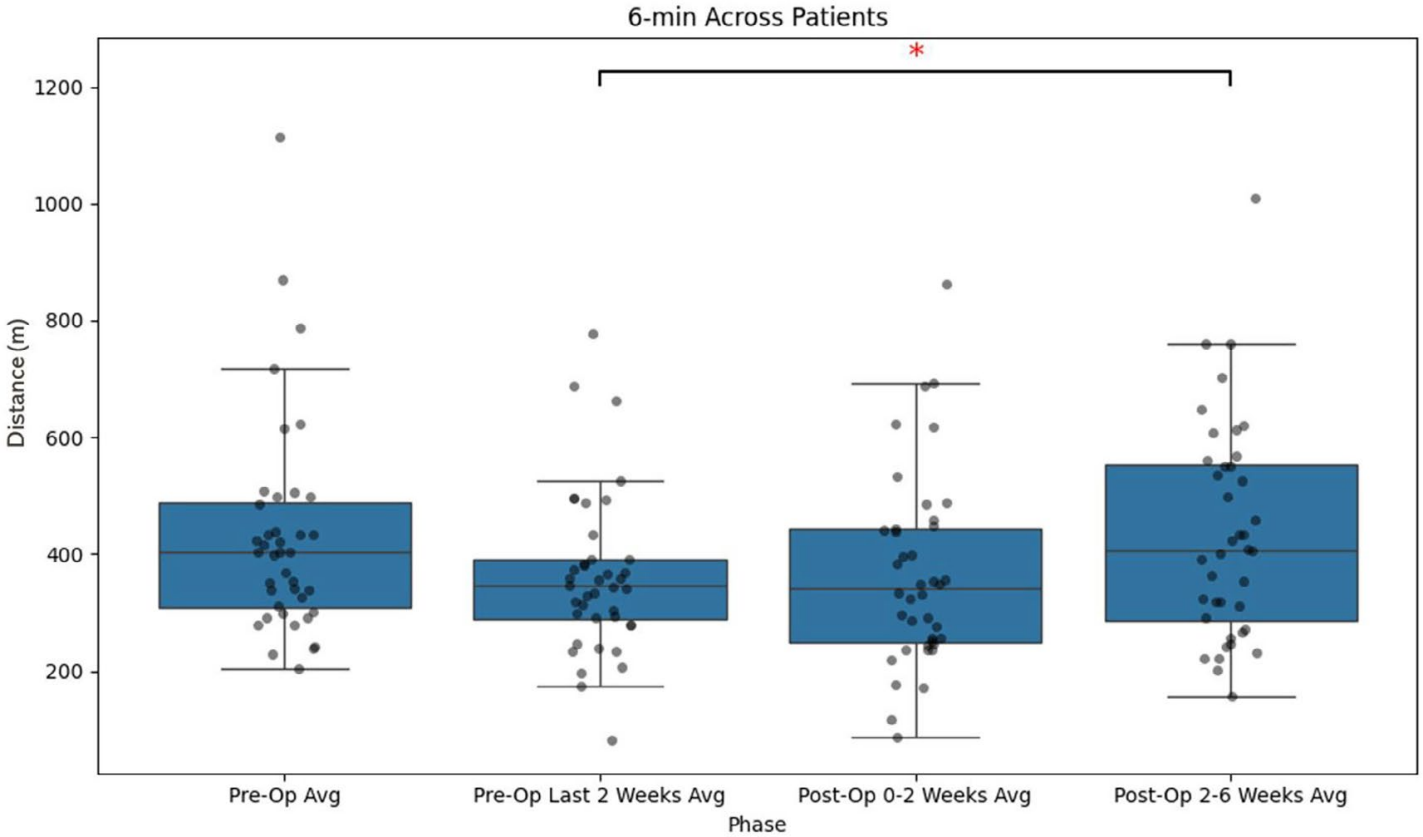
Boxplot showing the distribution of maximum daily distances in the 6MWT across different time periods. The box represents the interquartile range (IQR), with the lower and upper edges corresponding to the 25th and 75th percentiles. The horizontal line within the box indicates the median. Whiskers extend to the most extreme data points within 1.5 times the IQR, while individual dots represent outliers beyond this range. * indicates statistical significance (*p* < 0.05). Distance (*m*) represents iPhone‐estimated walking/running distance in meters.

The 6MWT demonstrated a preoperative median of 403 m (IQR, 309–489), which declined to 345 m (IQR, 288–391) in the final 2 weeks before surgery. Early postoperative performance (0–2 weeks) remained at a similar level (340 m; IQR, 249–444). By the late postoperative period (2–6 weeks), full recovery was observed (407 m; IQR, 287–554). Improvement from the last 2 weeks preoperatively to the late postoperative period reached statistical significance (*p* < 0.05), whereas the decline from the preoperative baseline to the last 2 weeks preoperatively trended toward significance (*p* = 0.07) (Figure 2).

### 3.6 Daily Step Counts

**FIGURE 3:**
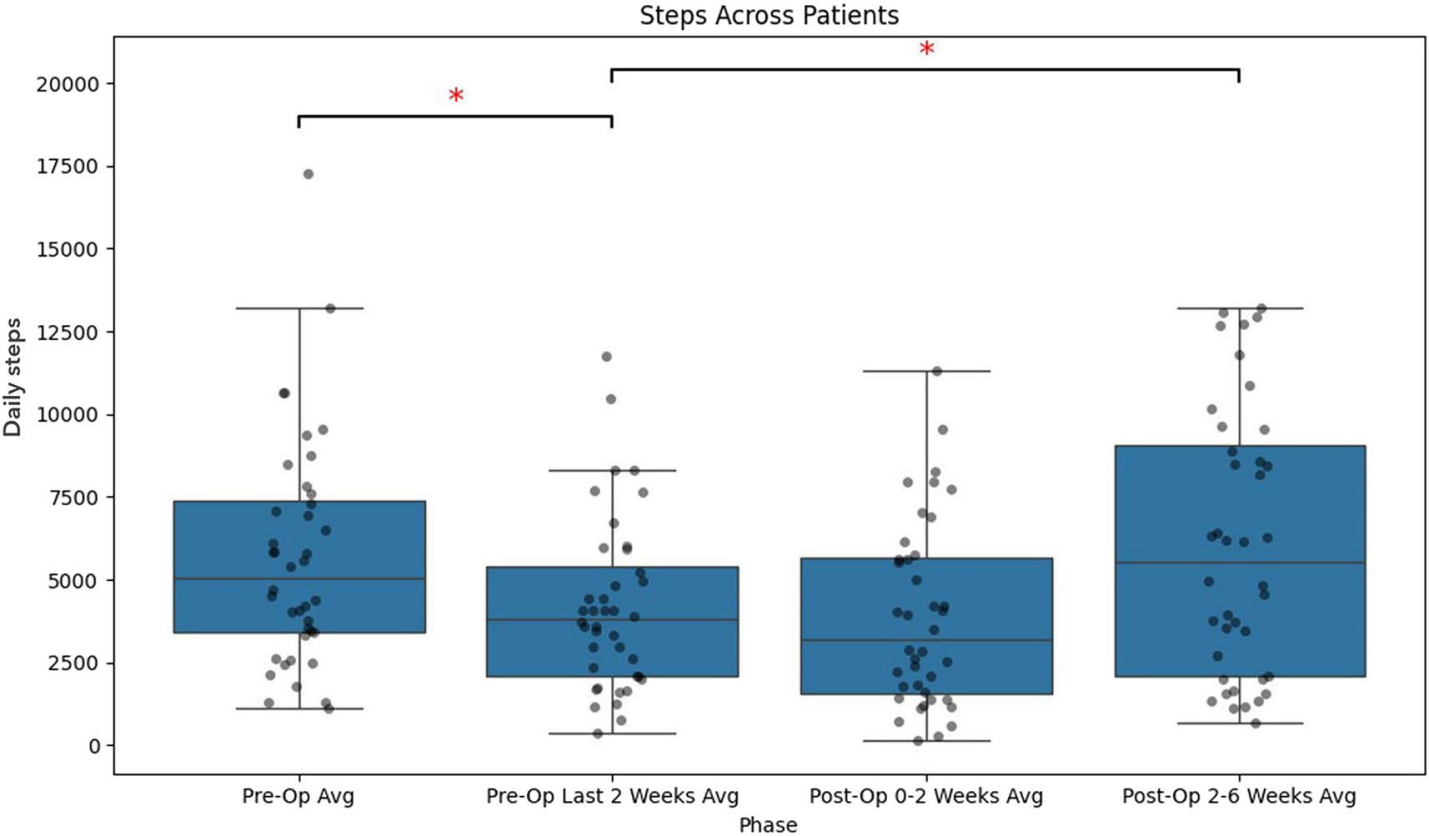
Boxplot illustrating daily step counts across time periods. The box represents the interquartile range (IQR), with the horizontal line indicating the median. Whiskers extend to the most extreme data points within 1.5 times the IQR, and individual dots represent outliers. * indicates statistical significance (*p* < 0.05). Daily steps represent iPhone‐estimated daily step count.

Daily step counts declined from a preoperative median of 5030 (IQR, 3390–7366) steps to 3825 (IQR, 2091–5390) steps in the final 2 weeks before surgery, representing a significant reduction (*p* < 0.05). Early postoperative activity (0–2 weeks) remained at a similarly reduced level (3177 steps; IQR, 1568–5659). By the late postoperative period (2–6 weeks), step counts increased significantly to 5538 (IQR, 2073–9047) compared with the final 2weeks preoperatively (*p* < 0.05; Figure 3).

### 3.7 Correlation Between Smartphone‐Derived Mobility Metrics

**FIGURE 4:**
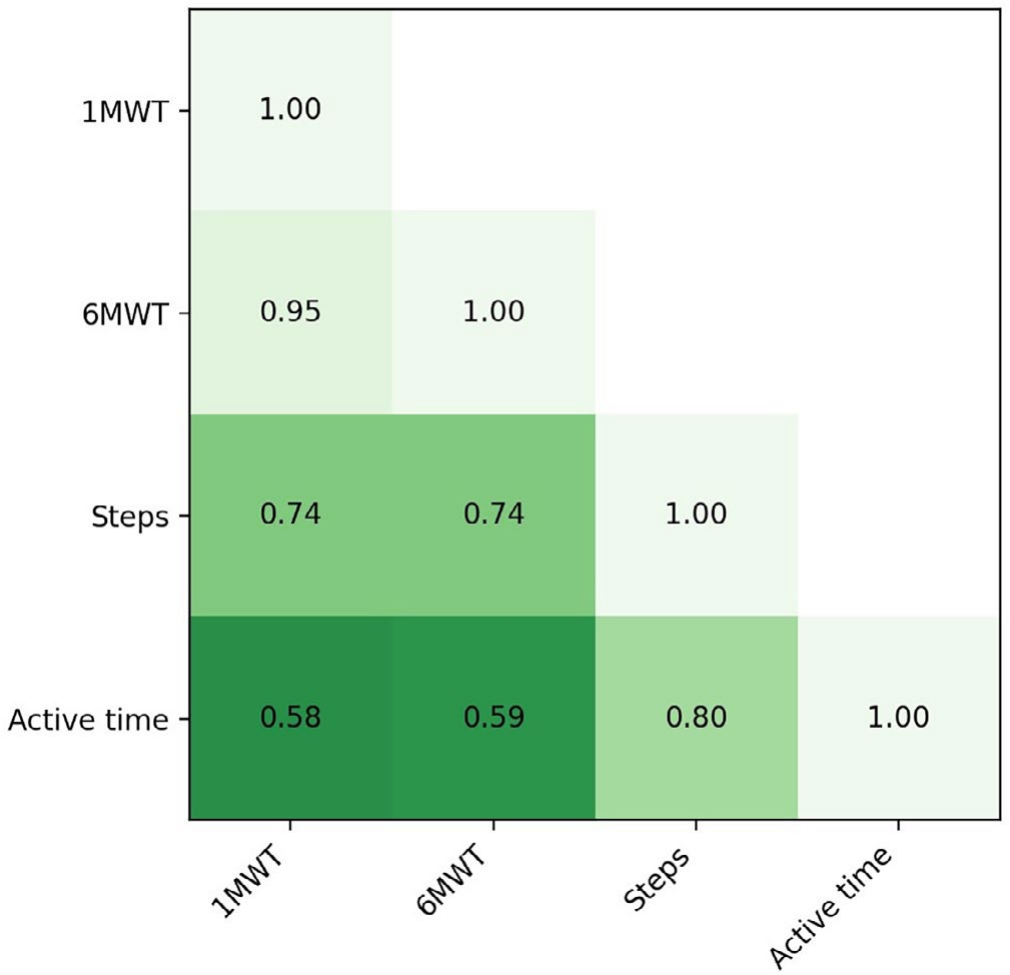
Pearson correlations between smartphone‐derived mobility metrics.

Across all available patient days (6 months preoperatively to 6 weeks postoperatively; *N* = 7955), the simulated 1 min and 6 min maxima were strongly correlated (Pearson *r* = 0.95, *p* < 0.001). Correlations with daily step count were moderate to strong (1MWT *r* = 0.74; 6MWT *r* = 0.74; both *p* < 0.001), while correlations with the active time proxy were smaller (1MWT *r* = 0.58; 6MWT *r* = 0.59; both *p* < 0.001). Daily step count and active time were strongly correlated (*r* = 0.80, *p* < 0.001; Figure 4). Because the dataset contains repeated observations per patient, these pooled correlations are descriptive.

### 3.8 Variability in Walking Performance, Step Counts, and Active Time

The CV was lowest for the 1MWT and 6MWT across all time periods. At baseline, the CV for the 1MWT and 6MWT was 46.1% (SD = 12.9) and 44.3% (SD = 14.5), respectively, while steps and active time showed higher variability at 70.7% (SD = 23.6) and 59.3% (SD = 22.4). A similar pattern was observed in the last 2 weeks preoperatively, where the CV for the 1MWT and 6MWT remained lower (43.1% and 42.5%) compared to steps (66.5%) and active time (53.8%).

**FIGURE 5:**
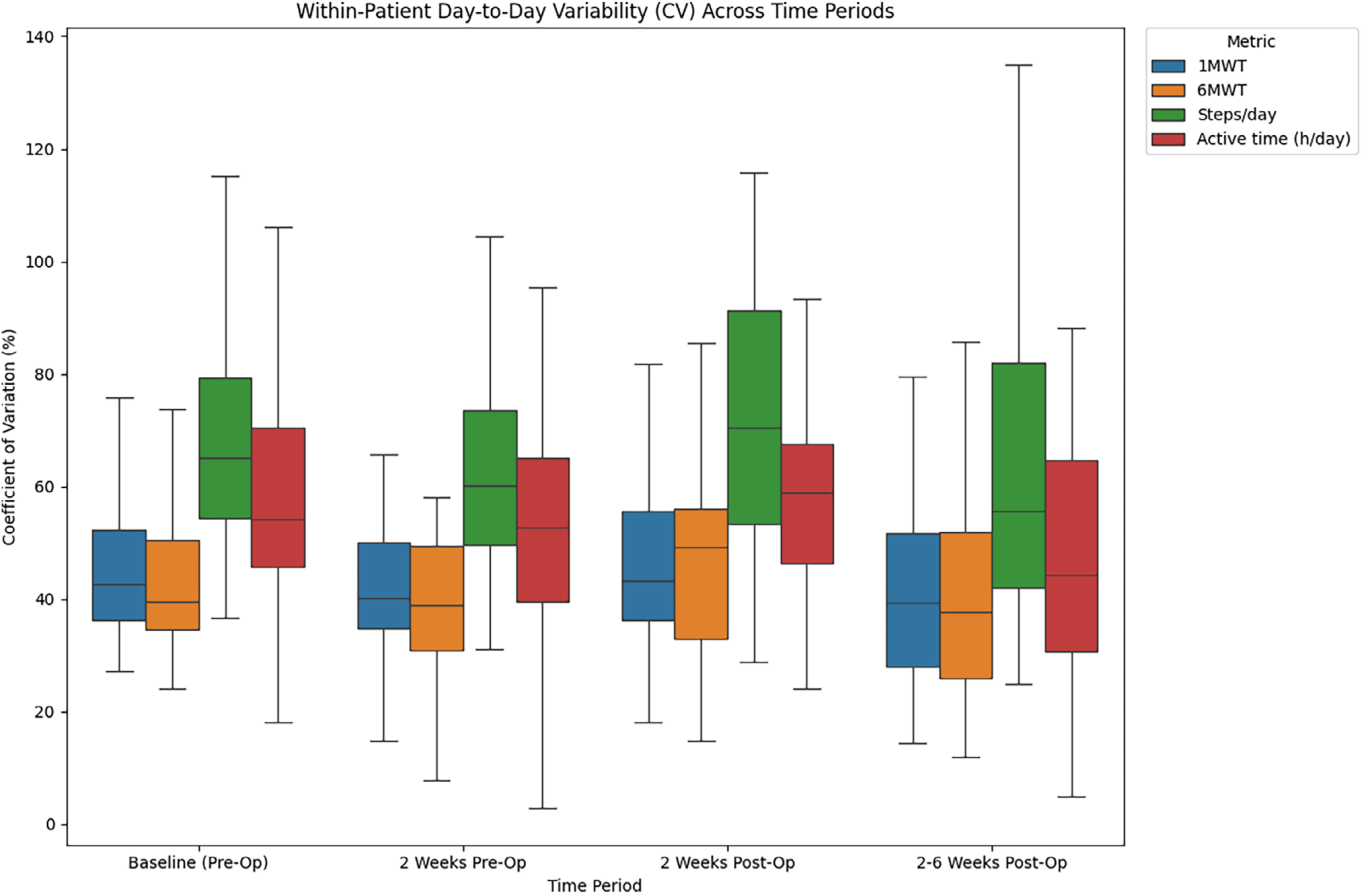
Boxplot illustrating the CV across time periods for each metric. The box represents the interquartile range (IQR), with the horizontal line indicating the median. Whiskers extend to the most extreme data points within 1.5 times the IQR.

Postoperatively, variability remained higher for steps and active time. In the early postoperative period (0–2 weeks), the CV increased for all metrics but was most pronounced for steps (73.2%, SD = 26.6) and active time (60.3%, SD = 32.5). The 1MWT and 6MWT showed a moderate increase in CV at 48.2% and 49.1%, respectively. In the late postoperative period (2–6 weeks), variability slightly decreased across all metrics, with the lowest values observed again in the 1MWT (40.4%) and 6MWT (39.4%), while steps and active time remained higher at 62.6% and 47.4%, respectively. These patterns are illustrated in Figure 5.

## 4 Discussion

This study evaluated the feasibility of smartphone‐derived activity data to assess functional recovery in patients undergoing lumbar decompression or discectomy. The findings demonstrate that shorter interval‐based measures, such as 1MWT and 6MWT, yield more stable and less variable approximations of functional capacity than daily step counts and active time. These shorter‐duration measures exhibited lower variability (see Figure 5). It is possible that they better reflect a patient's ability to mobilize, whereas cumulative daily metrics may be more influenced by day‐to‐day variation in activity patterns. However, this remains to be confirmed in further studies. Importantly, step counts, the 1MWT, and 6MWT all showed statistically significant improvements between the last 2 weeks preoperatively and the 2–6‐week postoperative period, highlighting their potential as objective markers of recovery (Figures 1, 2, 3). While step counts also improved significantly, they demonstrated greater variation compared to the 1MWT and 6MWT, suggesting that they may better reflect an approximation of performance rather than functional capacity.

The strong correlation between the simulated 1MWT and 6MWT indicates that both short interval maxima capture closely related peak walking behavior in daily life. In contrast, only moderate correlations with daily step count and active time suggest that cumulative daily metrics reflect additional external influences and daily patterns (e.g., lifestyle habits and smartphone carrying behavior) and are not interchangeable with short interval maxima. Because these correlations are pooled across repeated observations per patient, they should be interpreted descriptively.

Consistent with the recovery patterns described by Haddas et al. [4] the data exhibited three distinct phases: a decline in activity during the preoperative period as the disease burden intensified, low activity levels immediately after surgery, and gradual improvement during the recovery phase (illustrated in Figures 1, 2, 3). Variability analysis further supported the robustness of the 1MWT and 6MWT, which demonstrated lower CV across all time periods compared to step counts and active time (Figure 5). The CV for these short‐interval tests ranged from approximately 40%–50%, while step counts and active time exhibited greater fluctuations, often exceeding 60%–70%. These findings suggest that the shorter interval‐based measures may be less influenced by external factors and therefore provide a more stable approximation of capacity‐related aspects within real‐world functional performance.

For clinical context, we benchmarked the magnitude and perioperative change of our derived daily maximum 6 min distance against prospective cohorts using standardized 6MWT protocols in degenerative lumbar surgery populations, where mean 6MWT distance increased from 378–401 m preoperatively to 490–495 m at a single 6‐week postoperative follow‐up visit [23, 24, 25]. In contrast, our derived daily maximum 6 min distance reflects free living metrics and was summarized as the median across many days within postoperative weeks 2–6, with median values of 403 m at baseline and 407 m in weeks 2–6. This value may therefore be lower than a one‐time motivated test performed at a fixed 6‐week visit, a time point that may coincide with later functional recovery.

Stienen et al. [14] reported a similar postoperative recovery pattern using consumer‐grade wearable accelerometers, including analysis of both fusion and decompression groups. Their study observed a 71% reduction in step counts during the first postoperative week, followed by a partial recovery in the second and fourth weeks. However, no significant improvement beyond baseline was detected within the first postoperative year. In contrast, this study found that recovery occurred more rapidly, with significant improvements in functional capacity already evident between weeks 2 and 6 postoperatively.

The discrepancy between these results and those of Stienen et al. [14] may be due to differences in study design and data collection methods. The inclusion of both a long‐term (6‐month) preoperative baseline and a short‐term (2‐week) preoperative period in this study provided a more nuanced understanding of activity trajectories. Additionally, Stienen et al. [14] reported no sustained improvement beyond the preoperative level, which contrasts with the findings of continuous postoperative improvement in this study. This discrepancy may be explained by differences in surgical techniques, rehabilitation protocols, or patient demographics. Further research is needed to explore these differences in detail.

In their study, Stienen et al. [14] found no significant correlation between daily step counts and PROMs, a finding supported by other studies [6, 26], and something that could be further investigated in a follow‐up study in our cohort. However, they observed a strong association between step counts and depression, highlighting the complex interplay between physical activity, subjective health perceptions, and mental health in spine surgery patients. Such findings underscore the limitations of relying solely on a single parameter, such as daily step counts, to evaluate recovery trajectories.

Smartphone carrying behavior was analyzed using daily active time as a surrogate measure to assess the reliability of step count and walking distance data. The analysis showed that patients carried their phones for approximately 2.38 h per day preoperatively, which remained stable postoperatively at 2.26 h in the 2–6‐week recovery phase. These results align with findings from the ProPASS consortium, which reported consistent smartphone usage patterns across diverse populations [27]. The stable carrying behavior suggests that smartphone‐derived metrics reliably reflect activity trends, particularly for shorter‐duration walking assessments.

Step counts and active time reflect real‐world mobility but are susceptible to variation from lifestyle and phone‐carrying habits. The 1MWT and 6MWT are walking assessments that may be less affected by these sources of variability; however, our data do not directly substantiate this. We therefore present it as a testable hypothesis that should be empirically validated in future studies. Combined monitoring could therefore help interpret recovery trajectories: improvements in daily maxima with persistently low step counts may indicate improving peak walking ability with limited activity engagement, whereas increasing step counts without concurrent improvement in daily maxima may reflect greater activity participation without corresponding gains in peak walking ability. These interpretations are exploratory and should be evaluated further before informing clinical decision making.

This study has several strengths that contribute to its clinical relevance. Patients were unaware of their inclusion in the study until the 6‐week follow‐up, reducing the risk of the Hawthorne effect and enhancing data validity. The widespread availability of smartphones equipped with motion sensors allows for cost‐effective and large‐scale activity monitoring without the need for specialized wearable devices. The use of shorter interval‐based measures reduces variability and offers a more accurate representation of patients' ability to mobilize, as opposed to step counts, which may be more indicative of overall activity levels. The ability to collect continuous, day‐to‐day activity data provides a more comprehensive understanding of recovery trajectories than traditional outcome measures, which rely on predefined follow‐up intervals and may support earlier detection of atypical rehabilitation trajectories.

Despite its strengths, this study has several limitations. While interval‐based measures reduce bias from phone‐carrying habits, step count and active time may still be affected by whether and how patients carried their phones. Phone placement (e.g., pocket vs. bag) may introduce measurement bias. In addition, iPhone‐based mobility metrics depend on iPhone carriage. Intermittent carriage or non‐wear may underestimate total mobility; in retrospective smartphone data, non‐wear cannot be reliably distinguished from true inactivity, and peak walking episodes may be missed. Therefore, days with zero or very low recorded activity were not excluded or imputed. Generalizability may be limited to degenerative lumbar decompression and discectomy, as other procedures can involve different baseline impairment, postoperative restrictions, and recovery timelines.

Our cohort was predominantly ASA 1 to 2 (36/41), and feasibility, interpretability, and clinical utility in patients with higher comorbidity burden or lower baseline activity require dedicated evaluation. An additional potential advantage of short interval daily metrics is that they can be derived from brief walking episodes and may therefore be feasible for monitoring recovery trajectories even in cohorts with higher comorbidity burden, lower baseline mobility, or shorter smartphone carriage duration. This hypothesis requires confirmation in future studies. Inclusion required iPhone use, which may introduce a potential selection bias and limits generalizability.

Postdischarge physiotherapy and rehabilitation exposure and smartphone carrying habits were not controlled, and their potential influence on the activity metrics was not modeled. Future studies should explore methods to standardize data collection further:

In addition, the extent to which the observed 1MWT and 6MWT values correspond to actual functional capacity (maximum potential ability) under standardized test conditions is unknown. The derived measures should therefore be interpreted as approximations derived from real‐world performance data rather than direct measures of functional capacity.

The lack of a direct comparison with standardized 1MWT and 6MWT assessments precludes validation of these metrics as measures of functional capacity.

Importantly, we did not perform correlation or association analyses between smartphone‐derived metrics and PROMs. This represents a major limitation, leaving the relationship between passive smartphone monitoring and conventional clinical outcomes unresolved. Future research should explicitly assess associations between passive smartphone metrics, objective clinical assessments (e.g., gait analysis), and PROMs in larger, more diverse cohorts with varied spine pathologies and surgical interventions. Machine learning algorithms could subsequently be leveraged to predict individual recovery trajectories and personalize postoperative care [28, 29].

This study demonstrates that smartphone‐derived activity metrics, particularly 1MWT and 6MWT intervals, offer a feasible and objective method to monitor recovery following spine surgery. By distinguishing between cumulative real‐world performance metrics and short‐interval, capacity‐oriented measures derived from daily activity data, this approach provides valuable insights into patient rehabilitation. The ability to collect continuous activity data through smartphones presents a scalable, cost‐effective method for monitoring postoperative recovery and optimizing patient care.

## Conflicts of Interest

The authors declare no conflicts of interest.

## Data Availability

The data that support the findings of this study are available on request from the corresponding author. The data are not publicly available due to privacy or ethical restrictions.
